# Revisiting Biomarker-Guided Therapy in EGFR-Mutant Non-Small Cell Lung Cancer with High PD-L1 Expression

**DOI:** 10.3390/ijms27073294

**Published:** 2026-04-05

**Authors:** Nuri Park, Yejin Cho, Hong-Mei Zheng, Woo Kyung Ryu, Kyung Hee Jung, Jun Hyeok Lim

**Affiliations:** 1 Department of Biomedical Sciences, College of Medicine, and Program in Biomedical Science & Engineering, Inha University, Incheon 22332, Republic of Koreayejinvely@inha.edu (Y.C.); 325153@inha.ac.kr (H.-M.Z.);; 2 Division of Pulmonology, Department of Internal Medicine, Inha University Hospital, Inha University College of Medicine, Incheon 22332, Republic of Korea

**Keywords:** EGFR-mutant non-small cell lung cancer, PD-L1 expression, EGFR tyrosine kinase inhibitors, primary resistance, tumor heterogeneity, immune microenvironment, precision oncology, combination therapy

## Abstract

Epidermal growth factor receptor (EGFR)-mutant non-small cell lung cancer (NSCLC) has historically been regarded as a therapeutically uniform entity, characterized by marked sensitivity to EGFR tyrosine kinase inhibitors (TKIs) and limited responsiveness to immune-checkpoint inhibitors (ICIs). However, accumulating clinical and translational data suggest heterogeneity within EGFR-mutant NSCLCs. In particular, patients whose tumors express high levels of programmed death-ligand 1 (PD-L1) consistently experience inferior outcomes with EGFR-TKI monotherapy, including earlier progression and reduced response durability, even with third-generation EGFR-TKIs. This review synthesizes clinical, molecular, and immunologic evidence supporting the hypothesis that EGFR-mutant NSCLC with high PD-L1 expression may represent a biologically distinct phenotype. Key findings include data from retrospective cohorts, real-world analyses, and translational studies showing high PD-L1 expression to be associated with attenuated oncogene addiction, increased genomic complexity, tumor cell plasticity, and a dysfunctional but non-quiescent immune microenvironment. Notably, in this context, PD-L1 expression does not reliably predict benefit from ICIs but, rather, serves as a marker of aggressive tumor biology and early resistance to EGFR-TKI therapy. Lastly, we discuss the therapeutic implications of these observations, outlining the rationale for biomarker-informed, risk-adapted treatment strategies, including EGFR-TKI-based combinations, while emphasizing the need for careful integration of immunotherapy and prospective validation.

## 1. Introduction

Activating epidermal growth factor receptor (EGFR) mutations represent a molecularly distinct subset of non-small-cell lung cancer (NSCLC) that accounts for approximately 10–15% of cases in Western populations and up to 40–50% of NSCLCs in East Asian patients. The identification of such EGFR mutations and the subsequent development of EGFR tyrosine kinase inhibitors (TKIs) have fundamentally transformed the treatment landscape for advanced NSCLC. In particular, multiple landmark randomized trials have consistently found that patients with EGFR-mutant disease show superior response rates and prolonged progression-free survival (PFS) with EGFR-TKIs compared with platinum-based chemotherapy, firmly establishing targeted therapy as the standard first-line treatment for this NSCLC subtype [1,2,3,4].

In parallel, immune-checkpoint inhibitors (ICIs) targeting the programmed death 1 (PD-1)/programmed death-ligand 1 (PD-L1) axis have revolutionized the management of EGFR-wild-type NSCLC. Accordingly, PD-L1 expression, assessed by immunohistochemistry, is widely used as a predictive biomarker to guide the administration of immunotherapy in clinical practice. However, patients with EGFR-mutant NSCLC have consistently shown limited benefit from ICI monotherapy, even those with tumors expressing high levels of PD-L1, highlighting a fundamental disconnect between PD-L1 expression and immunotherapy efficacy in this molecular context [5,6,7].

Accumulating clinical and translational data further suggest that beyond attenuated sensitivity to EGFR-TKIs, EGFR-mutant tumors exhibiting high PD-L1 expression may represent an emerging phenotype with distinct biological characteristics and unfavorable clinical outcomes, thus presenting a biological and therapeutic paradox. These observations challenge the conventional view of EGFR-mutant NSCLCs as a homogeneous, uniformly TKI-sensitive population, raising important questions regarding biomarker interpretation and optimal treatment strategies. In this review, we critically examine the current evidence supporting the classification of EGFR-mutant NSCLC with high PD-L1 expression as a distinct biological and clinical subset and discuss the implications for biomarker-guided precision therapy.

## 2. Canonical Paradigm: EGFR-Mutant NSCLC as a TKI-Sensitive and Immunologically Cold Tumor

The canonical understanding of EGFR-mutant NSCLC is grounded in the framework of oncogene addiction, wherein tumors harboring common sensitizing EGFR alterations, most notably exon 19 deletions and the L858R mutation in exon 21, depend on EGFR signaling to sustain proliferation and survival. Clinically, this dependence is reflected by the rapid and often dramatic tumor regressions observed after initiation of EGFR tyrosine kinase inhibitor (EGFR-TKI) therapy, yielding response rates and PFS consistently superior to those obtained with platinum-based chemotherapy in first-line randomized trials [1,2].

**Figure 1:**
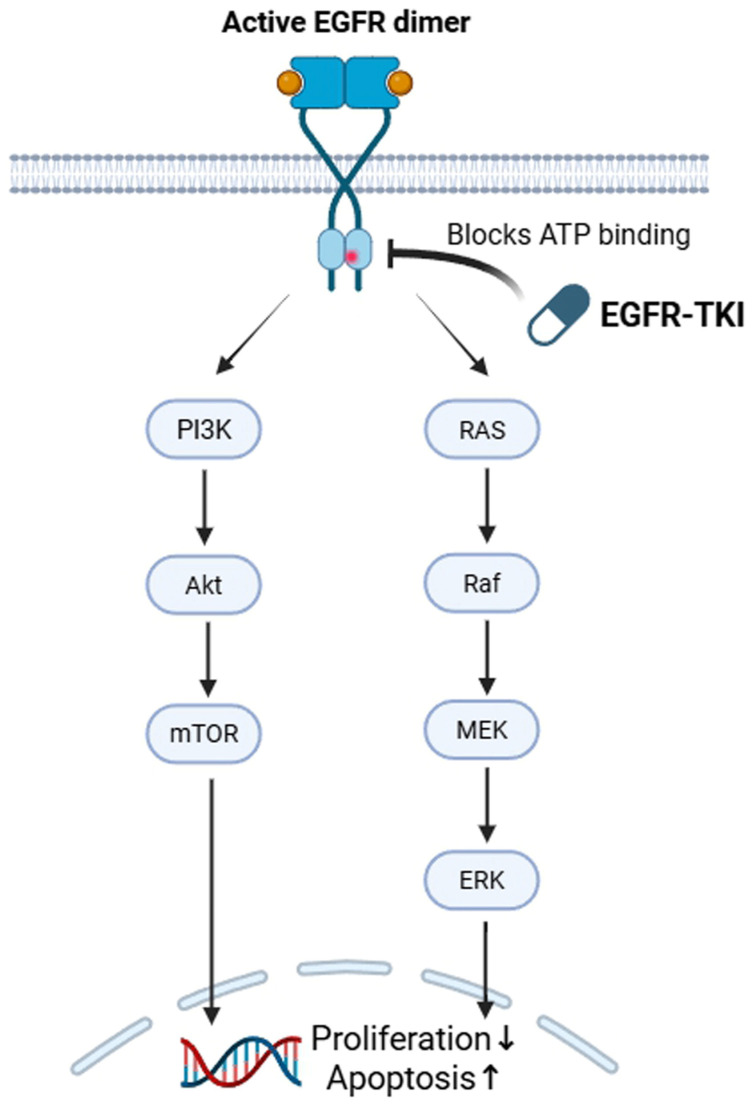
EGFR signaling and mechanism of action of EGFR tyrosine kinase inhibitors in EGFR-mutant non-small-cell lung cancer. Activating EGFR mutations results in constitutive receptor signaling and downstream activation of the PI3K/AKT/mTOR and RAS/RAF/MEK/ERK pathways, promoting tumor cell proliferation and survival. EGFR-TKIs inhibit EGFR kinase activity by blocking ATP binding, thereby suppressing these signaling pathways. This inhibition leads to reduced proliferation and increased apoptosis, reflecting the dependence of EGFR-mutant tumors on oncogenic EGFR signaling.

EGFR tyrosine kinase inhibitors (EGFR-TKIs) exert their therapeutic effects by selectively inhibiting the kinase activity of mutant EGFR, thereby blocking downstream signaling pathways essential for tumor cell proliferation and survival. Upon ligand binding, EGFR activation triggers multiple intracellular cascades, including the phosphoinositide 3-kinase (PI3K)/AKT and mitogen-activated protein kinase (MAPK)/extracellular signal-regulated kinase (ERK) pathways. EGFR-TKIs disrupt these signaling networks, leading to cell cycle arrest and apoptosis in EGFR-dependent tumor cells (Figure 1). First- and second-generation EGFR-TKIs inhibit EGFR through reversible or irreversible binding to the ATP-binding site, whereas third-generation inhibitors, such as osimertinib, are designed to selectively target both sensitizing EGFR mutations and the T790M resistance mutation while sparing wild-type EGFR. Despite initial efficacy, resistance to EGFR-TKIs inevitably develops through diverse mechanisms, including secondary EGFR mutations, activation of bypass signaling pathways, and phenotypic transformations such as epithelial-to-mesenchymal transition.

The development of third-generation EGFR-TKIs provided further support for this targeted-therapy paradigm. Osimertinib, in particular, which was developed to address resistance mechanisms, such as T790M, has shown robust clinical activity against treatment-naïve EGFR-mutant disease. In the Phase III FLAURA trial, osimertinib improved PFS compared with first-generation EGFR-TKIs and subsequently provided an overall survival advantage, cementing osimertinib as a preferred first-line standard treatment [3,4]. In addition to this systemic efficacy, osimertinib’s ability to treat and prevent central nervous system progression has reinforced the perception that EGFR-mutant NSCLC is optimally managed by EGFR pathway inhibition rather than by immunomodulatory strategies.

In addition to its noted sensitivity to targeted therapy, EGFR-mutant NSCLC has traditionally been perceived as an immunologically “cold” tumor type. That is, compared with smoking-associated EGFR-wild-type tumors, EGFR-mutant cancers typically have a lower tumor mutational burden and a correspondingly reduced neoantigen repertoire, features strongly associated with diminished sensitivity to PD-1 blockade across NSCLC populations [8]. Consistent with this model, EGFR-mutant NSCLCs frequently show limited infiltration by activated cytotoxic T lymphocytes and a tumor microenvironment skewed toward immunosuppression. Collectively, these features may blunt endogenous anti-tumor immunity and limit the capacity of PD-1 or PD-L1 blockade to restore effective immune surveillance [9].

Clinically, the “cold tumor” paradigm is reinforced by consistent outcome data showing inferior activity of ICIs against EGFR-mutant NSCLC. One meta-analysis focused on metastatic EGFR-mutant NSCLC reported poor outcomes overall following checkpoint blockade [5]. Similarly, a pivotal retrospective analysis revealed low objective response rates to PD-1 or PD-L1 blockade among patients with EGFR mutations and anaplastic lymphoma kinase (ALK) rearrangements, accompanied by low rates of concurrent PD-L1 expression and CD8-positive tumor-infiltrating lymphocytes, features consistent with an immunologically inert microenvironment [6]. Although heterogeneity exists within EGFR-mutant disease, including differences among EGFR mutation subtypes, overall responses to ICIs remain muted compared with those observed for EGFR-wild-type NSCLC [7].

Mechanistically, EGFR-driven tumors have also been shown to engage immune escape programs involving the PD-1/PD-L1 axis. For example, PD-1 pathway activation was found to promote immune evasion in EGFR-mutant lung tumor models, suggesting that oncogenic EGFR signaling can shape the tumor-immune interface in ways that are unfavorable for checkpoint blockade [10]. These biologic observations are consistent with the aforementioned clinical findings showing that EGFR-mutant NSCLC generally derives limited benefit from PD-1 or PD-L1 inhibitors, even when PD-L1 is detectable. Thus, in this context, PD-L1 expression does not necessarily reflect a productive, immunotherapy-responsive anti-tumor immune response [5,6,7].

**Figure 2:**
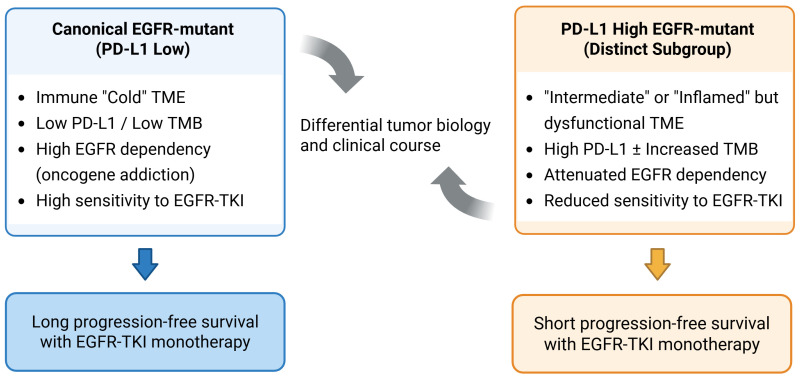
Conceptual schematic illustrating the heterogeneity of EGFR-mutant NSCLC and the proposed biological differences associated with high PD-L1 expression. This figure summarizes findings from multiple clinical and translational studies and is not based on a single dataset. Canonical EGFR-mutant NSCLC with low PD-L1 expression is characterized by strong EGFR dependency, an immunologically “cold” tumor microenvironment, and durable responses to EGFR tyrosine kinase inhibitor (TKI) monotherapy. In contrast, EGFR-mutant tumors expressing high levels of PD-L1 represent a distinct subgroup with attenuated EGFR dependency, characterized by the presence of immune cell infiltration alongside impaired cytotoxic T-cell activity, enrichment of immunosuppressive signaling pathways, and reduced sensitivity to EGFR-TKI monotherapy, resulting in poor progression-free survival (PFS).

Based on the above findings, contemporary clinical practice has long favored EGFR-targeted therapy as the primary first-line approach for EGFR-mutant NSCLC, with ICIs positioned as later-line options or reserved for specific circumstances. This therapeutic sequencing reflects the prevailing consensus that EGFR-mutant NSCLC is both highly sensitive to EGFR-TKIs and intrinsically less immunogenic, rendering ICI monotherapy unreliable in most cases regardless of PD-L1 expression. However, emerging clinical and translational data now suggest that EGFR-mutant NSCLC is not a homogeneous disease but, rather, that there is a discrete subset with high PD-L1 expression that exhibits distinct biological characteristics and clinical behavior (Figure 2).

## 3. Clinical Observations: High PD-L1 Expression Identifies an Atypical EGFR-Mutant Subgroup with Suboptimal Response to TKIs

Despite the overall success of EGFR-TKIs against NSCLC, clinical data increasingly show heterogeneous outcomes among patients with EGFR-mutant forms of the disease. Moreover, within the past decade, numerous retrospective analyses, real-world cohorts, and correlative studies have converged on the consistent observation that high tumoral PD-L1 expression is associated with diminished and less durable benefit from EGFR-TKI monotherapy. Thus, there is mounting evidence for an atypical subgroup within EGFR-mutant NSCLC characterized by elevated levels of PD-L1.

Early clinical signals suggesting the existence of a PD-L1-high subgroup emerged from treatment-naïve EGFR-mutant lung cancer cohorts receiving first- and second-generation EGFR-TKIs. One of the earliest studies to highlight this phenomenon reported that strong PD-L1 expression in EGFR-mutant NSCLC was associated with de novo resistance and poor response to EGFR-TKIs, introducing the concept of PD-L1-linked primary resistance in this molecular subtype [11]. Similarly, strong PD-L1 expression in advanced EGFR-mutant lung adenocarcinoma pretreatment samples was associated with a markedly higher incidence of primary resistance and significantly shorter PFS compared with low or absent PD-L1 expression [12]. Notably, these findings were based on expression in baseline specimens, indicating that the PD-L1 levels observed reflected intrinsic tumor characteristics rather than therapy-induced adaptation.

Subsequent studies across different institutions and clinical settings reinforced the above observations. As one example, clinical analyses of EGFR-mutant lung adenocarcinoma patients treated with EGFR-targeted therapy consistently showed inferior PFS and, in some cohorts, reduced overall survival among patients with high PD-L1 expression relative to those with lower expression, even when baseline clinicopathologic features were broadly comparable [13,14]. These findings suggest that PD-L1 expression captures prognostic information not fully explained by conventional clinical variables.

Refinement of PD-L1 stratification further strengthened the clinical signal for the observed associations. That is, when PD-L1 expression was analyzed based on tumor proportion score (TPS) thresholds, strong expression, typically defined as a score ≥ 50%, was repeatedly associated with poor EGFR-TKI efficacy. Several EGFR-mutant lung cancer cohorts further showed a graded relationship between increasing PD-L1 expression and progressively shorter PFS, supporting a continuum rather than a binary association between PD-L1 levels and treatment outcome [15].

The transition to third-generation EGFR-TKIs as the first-line standard of care allowed investigators to evaluate whether more potent EGFR inhibition would improve efficacy against EGFR-mutant lung cancers expressing high levels of PD-L1. However, results from contemporary real-world studies revealed that even these drugs could not overcome the adverse association with PD-L1 expression. In patient cohorts receiving first-line osimertinib, high PD-L1 expression remained associated with inferior PFS relative to lower PD-L1 levels, indicating that the negative prognostic impact of PD-L1 expression persists even in the modern treatment era [13,15]. This observation has also been substantiated in large translational real-world cohorts. One retrospective analysis of patients with EGFR-mutant lung adenocarcinoma treated with first-line osimertinib found that those with tumors with PD-L1 TPS ≥ 50% exhibited significantly shorter PFS and overall survival than those whose tumors exhibited lower PD-L1 expression. In contrast, objective response rates for the two groups were largely preserved. Integrated transcriptomic and immune profiling further revealed activation of interferon (IFN)-γ and the interleukin (IL)-6/Janus kinase (JAK)/signal transducer and activator of transcription 3 (STAT3) signaling axis, as well as enrichment of CD56^bright^ natural killer (NK) cells, in PD-L1-high tumors [16]. These observations suggest that elevated PD-L1 expression reflects a distinct biological state of EGFR-mutant tumors rather than a coincidental biomarker finding.

Correlative analyses from randomized trial populations have provided complementary insights. In an exploratory analysis, assessment of PD-L1 expression in untreated EGFR-mutant NSCLC samples revealed no clear enhancement of osimertinib benefit among PD-L1-high tumors compared with PD-L1-low tumors, indicating that high PD-L1 expression does not confer increased sensitivity to third-generation EGFR-TKIs [17].

Population-level data further corroborate these observations. One meta-analysis assessing levels of PD-L1 expression in EGFR-mutant NSCLC prior to treatment with EGFR-TKIs concluded that higher PD-L1 expression was consistently associated with poorer PFS, despite heterogeneity in PD-L1 assays, cutoff values, and study design [18]. More recent real-world cohorts using standardized TPS groupings have reported similar findings, noting that higher PD-L1 expression is associated with progressively worse outcomes of EGFR-TKIs [19]. Validating these observations, additional real-world and institutional cohort studies, including several analyses conducted in the first-line osimertinib era, have consistently reported inferior PFS among patients with EGFR-mutant NSCLC expressing high levels of PD-L1, further reinforcing the robustness of this association across diverse clinical settings (Table 1).

Beyond time-to-event outcomes, several studies have found that PD-L1-high EGFR-mutant tumors may exhibit a more aggressive clinical phenotype. In particular, these tumors have been associated with earlier disease progression and, in some reports, patterns suggestive of increased metastatic potential, including a higher likelihood of extrathoracic dissemination [11,14,19]. Consistent with these findings, broader profiling of immune-checkpoint proteins in EGFR-mutant NSCLC has revealed that PD-L1-high tumors often exhibit immune-related features associated with more aggressive clinical behavior rather than enhanced immunotherapy sensitivity [11]. Although such observations vary across cohorts and require prospective validation, existing profiling data support the hypothesis that PD-L1-high EGFR-mutant NSCLC behaves differently from the archetypal, indolent, EGFR-addicted tumor.

Taken together, the above findings suggest that high PD-L1 expression may identify a subset of EGFR-mutant NSCLC with reduced dependence on EGFR signaling and suboptimal benefit from EGFR-TKI monotherapy, although this appears to reflect a spectrum of risk rather than a clearly defined categorical subgroup. Importantly, in this context, PD-L1 expression functions primarily as a negative prognostic marker rather than as a predictor of benefit from immune-checkpoint blockade. This distinction places PD-L1-high EGFR-mutant disease in a therapeutic gray zone and highlights the need for alternative, risk-adapted treatment strategies, as discussed later in this review.

However, not all studies have demonstrated a consistent association between PD-L1 expression and EGFR-TKI outcomes. Analyses from prospective clinical trial datasets, including an exploratory analysis of the FLAURA study, have suggested that PD-L1 expression does not significantly influence progression-free survival in patients treated with osimertinib [17]. Similarly, meta-analytic data have indicated that PD-L1 expression may not be a reliable predictive or prognostic biomarker for EGFR-TKI efficacy, with pooled analyses showing no significant association with progression-free or overall survival [20]. Taken together, these findings highlight substantial heterogeneity across studies and suggest that the relationship between PD-L1 expression and EGFR-TKI outcomes remains context-dependent, heterogeneous across clinical settings, and not yet fully resolved.

**Table 1:** Published findings describing the clinical outcomes of EGFR-TKI treatment by PD-L1 expression in EGFR-mutant NSCLC.

Study	EGFR Mutations	PD-L1 Cutoff	TKI Generation	Sample Size	Outcomes (PD-L1 High vs. Low)	Conclusion
Su et al., 2018 [21]	Common (19del, L858R) ± uncommon)	TPS ≥ 50% (high)	1st-gen	N = 101	ORR 35.7% vs. 65–67% (PD-L1 high vs. weak/neg, *p* = 0.002); median PFS 3.8 vs. 9.5 mo (*p* < 0.001).	High PD-L1 = poor TKI response; significantly lower ORR and shorter PFS (indicative of de novo resistance) in PD-L1 ≥ 50% tumors.
Hsu et al., 2019 [12]	Common (no T790M)	TPS ≥1%, ≥25%, ≥50% (high)	1st/2nd-gen	N = 123	OR for primary resistance with PD-L1 high: 16.47 (vs. <50%, *p* = 0.008); median PFS ~1.6 vs. 7.3 mo if PD-L1 < 1%.	High PD-L1 = primary resistance. EGFR-mutant pts with PD-L1 high had ~5–16× higher odds of primary TKI resistance and significantly shorter PFS vs. PD-L1 low.
Yoon et al., 2020 [13]	Common (19del, L858R)	TPS ≥ 50% (high)	1st/2nd-gen	N = 131	ORR 43.5% vs. 75% (TPS ≥ 50% vs. <50%, *p* = 0.001); median PFS 8.3 vs. 16.9 mo (HR = 2.6, *p* = 0.004). OS not different overall. PD-L1-high group had less T790M at progression.	High PD-L1 = unfavorable outcomes. PD-L1 TPS ≥ 50% independently predicted shorter PFS and lower response rate.
Chang et al., 2021 [22]	Common (19del, L858R) ± uncommon	TPS ≥ 50% (high)	1st/2nd-gen	N = 114	No significant difference in PFS among TPS < 1%, 1–49%, and ≥50% groups (median PFS 13 mo for all, *p* > 0.05). OS and ORR not significantly different by PD-L1 status.	High PD-L1 = no impact. In this Taiwanese cohort, 9% had PD-L1 TPS ≥ 50%, and PD-L1 expression did not significantly affect PFS or OS with first-line TKI.
Kang et al., 2021 [15]	Common (19del, L858R)	TPS <1%, 1–49% ≥50% (high)	1st/2nd-gen	N = 108	Median PFS 7.1 vs. 12.7 vs. 14.7 mo (for TPS ≥ 50% vs. <1% vs. 1–49%; *p* < 0.01). PD-L1 TPS ≥ 50% remained an independent adverse factor (Cox HR = 2.1). No difference in acquired T790M rates by PD-L1 status.	High PD-L1 = poor PFS. PD-L1 TPS ≥ 50% was associated with significantly shorter PFS on first-line TKIs. Indicates a de novo resistance phenotype.
Liu et al., 2021 [14]	Common ± uncommon (G719, L861Q)	TPS ≥ 50% (high)	1st/2nd-gen	N = 186	Median PFS 6.6 vs. 13.0 mo (HR = 2.6, 95%CI: 1.6–4.2, *p* < 0.0001); median OS 11.5 vs. 32.9 mo (HR = 3.3, *p* < 0.0001) for TPS ≥ 50% vs. <50%. Differences were significant in multivariate analysis.	High PD-L1 = early progression and poor survival. Pts with PD-L1 TPS ≥ 50% had ~half the PFS and one-third the OS of those with TPS < 50% on 1st-gen TKIs. Strong prognostic effect regardless of ethnicity.
Peng et al., 2021 [18]	EGFR-mutant NSCLC (12 studies)	Various cutoffs	1st/2nd-gen	N = 991 total	Meta-analysis: PD-L1 high associated with significantly shorter PFS (pooled HR = 1.90, 95%CI: 1.16–3.10, *p* = 0.011). No significant OS difference overall (HR = 1.19, *p* = 0.07).	High PD-L1 = worse PFS. Across 991 pts, pretreatment PD-L1 overexpression predicted poorer PFS on EGFR-TKIs. (Pooled OS effect was not significant.)
Hsu et al., 2022 [23]	Common (19del, L858R)	TPS ≥ 50% (high)	3rd-gen (osimertinib first line)	N = 85	On first-line osimertinib: median PFS 9.7 vs. 26.5 mo for TPS ≥ 50% vs. <50% (HR = 0.19 with <50%, *p* = 0.009); median OS 25.4 mo vs. not reached (*p* = 0.021). ORR 79% overall.	High PD-L1 = poor outcome on osimertinib. PD-L1 TPS ≥ 50% was associated with markedly shorter PFS and inferior OS on first-line osimertinib.
Lei et al., 2023 [19]	Common + uncommon	TPS ≥ 50% (high)	Mixed 1st/2nd/3rd-gen	N = 117	ORR did not significantly differ by PD-L1 (ORR 51.9% vs. 43.2% vs. 64.0% for TPS ≥ 50% vs. <1% vs. 1–49%; *p* = 0.16). Median PFS 13.0 vs. 22.0 mo (for TPS ≥ 50% vs. <1%, *p* = 0.01). PD-L1 high was an independent risk factor for shorter PFS (multivariate HR not given). *TP53* co-mutation plus PD-L1 positivity identified a subgroup with the shortest PFS.	High PD-L1 = shorter PFS. In this Chinese cohort (with 50% on osimertinib), baseline PD-L1 TPS ≥ 50% was linked to significantly worse PFS, although differences in ORR were not significant.
Papazyan et al., 2024 [24]	Common + uncommon	TPS ≥ 50% (high)	3rd-gen (osimertinib first line)	N = 96	On first-line osimertinib: for TPS ≥ 50% vs. <50%, median PFS 9.3 vs. 17.5 mo (*p* = 0.044); median OS 14.3 vs. 26.0 mo (*p* = 0.025). PD-L1 ≥ 50% independently predicted shorter OS (HR = 2.61, *p* = 0.007); no significant baseline differences by PD-L1.	High PD-L1 = worse survival on osimertinib. PD-L1 TPS ≥ 50% was associated with significantly shorter PFS and OS on first-line osimertinib.
Yang et al., 2024 [25]	Acquired T790M+ (post-1st/2nd-gen)	Positive: TC ≥ 1% (vs. 0%)	3rd-gen (osimertinib 2L)	N = 134	Among T790M-positive pts on osimertinib, baseline PD-L1 status did not predict outcome. PFS 19.8 vs. 9.7 mo (TPS ≥ 1% vs. 0%, *p* = 0.67); no OS difference (42.9 vs. 33.5 mo, *p* = 0.91). TPS ≥ 50% subgroup likewise showed no PFS/OS difference. Multivariate Cox confirmed PD-L1 was not prognostic.	High PD-L1 = no impact in T790M setting. In pre-treated EGFR T790M+ pts receiving osimertinib, PD-L1 expression did not significantly affect PFS or OS. Laboratory models similarly showed no causative resistance from PD-L1 overexpression.
Zhang et al., 2025 [26]	Common	TPS ≥ 50% (high)	3rd-gen (osimertinib first line)	N = 182	On first-line 3rd-gen TKI: median PFS 13.6 vs. ~18.8 mo (TPS ≥ 50% vs. <50%, overall cohort, *p* = 0.026). TPS ≥ 50% was an independent risk factor (HR = 2.07, *p* = 0.011). Spatial effect: PD-L1 at primary tumor: ≥50% PFS 10.2 vs. 18–22 mo if lower (*p* < 0.001); PD-L1 in lymph node metastases showed no PFS difference.	High PD-L1 = reduced osimertinib efficacy. PD-L1 TPS ≥ 50% was associated with significantly shorter PFS on first-line osimertinib. Notably, PD-L1 status in primary lesions was predictive, whereas PD-L1 in metastatic nodes was not.
Alexander et al., 2026 [27]	Common (Ex19del, L858R)	TPS ≥ 50% (high)	3rd-gen (osimertinib first line)	N = 216	Real-world cohort: TPS ≥ 50% vs. <50%, adjusted HR = 3.03 for PFS (95%CI: 1.85–4.96, *p* < 0.001); median OS 40.4 vs. 57.0 mo (unadj.). PD-L1 high trended toward worse OS (only ≥75% cutoff reached multivariate significance). Meta-analysis (6 studies): Confirmed TPS ≥ 50% predicts shorter PFS (HR = 2.32, *p* = 0.018) and OS (HR = 2.38, *p* = 0.018) on first-line osimertinib.	High PD-L1 = 2–3× risk of progression on osimertinib. Across Australian multicenter data, PD-L1 TPS ≥ 50% was linked to significantly shorter real-world PFS and a non-significant trend toward shorter OS. A supporting meta-analysis corroborated a 2.3× higher risk of progression/death for TPS ≥ 50% pts on first-line osimertinib.

Abbreviations: CI, confidence interval; gen, generation; HR, hazard ratio; mo, months; ORR, overall response rate; OS, overall survival; pts, patients; TPS, tumor proportion score.

## 4. Biological Basis: Distinct Molecular and Immunologic Features Associated with Poor TKI Response

Programmed death-ligand 1 (PD-L1) is a key immune checkpoint molecule that plays a central role in regulating anti-tumor immune responses. By binding to its receptor PD-1 on activated T cells, PD-L1 inhibits T-cell proliferation, cytokine production, and cytotoxic activity, thereby facilitating immune evasion. PD-L1 expression can be regulated through both tumor-intrinsic and immune-mediated mechanisms. In EGFR-mutant NSCLC, oncogenic EGFR signaling has been shown to directly upregulate PD-L1 expression through downstream pathways such as PI3K/AKT and JAK/STAT. In parallel, inflammatory cytokines, particularly interferon-γ (IFN-γ), can induce PD-L1 expression as part of an adaptive immune resistance response within the tumor microenvironment. These dual regulatory mechanisms suggest that PD-L1 expression may reflect a complex interplay between tumor cell-intrinsic survival signaling and immune-mediated pressure. As a result, elevated PD-L1 expression in EGFR-mutant NSCLC may not necessarily indicate an effective anti-tumor immune response but rather a state of immune evasion or dysfunction.

Translational studies have begun to provide insight into why a subset of EGFR-mutant NSCLC tumors with high PD-L1 expression show attenuated and less durable benefit from EGFR-TKI monotherapy. Specifically, results from these studies suggest that PD-L1-high EGFR-mutant tumors exhibit several biological features that are inconsistent with a simple, uniform state of oncogene addiction, including increased molecular complexity, altered cell-state programs, and a distinct immune landscape. The evidence linking PD-L1 expression to EGFR-TKI resistance spans multiple levels, including hypothesis-generating clinical observations, mechanistic insights from preclinical models, and limited clinically validated data. These lines of evidence should be interpreted separately, as causal relationships remain incompletely established.

At the genomic level, clinical and translational studies have suggested that PD-L1-high EGFR-mutant tumors are associated with increased genomic instability and a higher frequency of co-occurring alterations, particularly those involving *TP53* and other cell cycle-related genes. Multiple clinical and translational analyses of EGFR-mutant NSCLC indicate that *TP53* co-mutations define a higher-risk subset with inferior outcomes following EGFR-TKI therapy and may reflect a more genomically complex tumor state that is less dependent on EGFR signaling alone [28,29]. Consistent with this model, genomic profiling of EGFR-sensitive tumors expressing high levels of PD-L1 has identified enrichment for pathway-level mutations, often including those affecting phosphoinositide 3-kinase (PI3K) signaling. These observations suggest that downstream signaling programs may be bypassed or altered at baseline in the PD-L1-high subgroup, thereby blunting the efficacy of EGFR-TKI monotherapy [30]. Thus, current findings support a mechanistic model in which elevated PD-L1 expression is a marker of broader oncogenic complexity, including co-mutations and pathway alterations that facilitate activation of alternative survival routes under EGFR inhibition [28,29,30].

In addition to genomic alterations, epithelial-to-mesenchymal transition (EMT) and related plasticity programs have long been implicated in intrinsic and early acquired resistance to EGFR-TKIs. Foundational work demonstrated that an epithelial versus mesenchymal phenotype predicts differential sensitivity to EGFR inhibition, with more mesenchymal states exhibiting reduced dependence on EGFR signaling and diminished drug sensitivity [31]. Subsequent studies in EGFR-mutant systems revealed that EMT can occur during acquired resistance to erlotinib, accompanied by phenotypic changes consistent with reduced reliance on the EGFR pathway [32]. In the context of PD-L1-high EGFR-mutant tumors, enrichment for partial EMT or hybrid cell-state signatures has been observed in preclinical and translational studies and may provide a biologic link between elevated PD-L1 levels, tumor invasiveness, and impaired durability of EGFR-TKI benefit. Although EMT is unlikely to be the sole mechanism mediating a reduced sensitivity to EGFR inhibition, this well-supported resistance program aligns with the more aggressive clinical behavior observed in some PD-L1-high cohorts [31,32].

Notably, recent mechanistic studies provide direct evidence that PD-L1 itself can drive intrinsic resistance to EGFR-TKIs. In particular, results from EGFR-mutant lung adenocarcinoma models have demonstrated that PD-L1 overexpression can promote autophagy and suppress apoptosis, thereby potentially reducing sensitivity to EGFR-TKIs. This effect was mediated by mitogen-activated protein kinase (MAPK)/extracellular signal-related kinase (ERK)-dependent signaling, establishing a cell-intrinsic PD-L1 autophagy axis that contributes to primary resistance. Thus, these findings offer a direct mechanistic explanation for the early progression observed in PD-L1-high EGFR-mutant tumors [33].

From an immunologic standpoint, PD-L1-high EGFR-mutant tumors have been reported to occupy an intermediate state between the canonical immunologically cold phenotype and an inflamed tumor microenvironment. Immune marker profiling across NSCLC samples bearing driver mutations has shown that EGFR-mutant tumors generally display immune features distinct from those associated with smoking-associated disease [34]. However, immune infiltration patterns vary across EGFR-mutant cases, indicating biologically meaningful heterogeneity [34]. Longitudinal analyses of pretreatment and post-progression samples further indicate that EGFR-TKI therapy can reshape the tumor-immune microenvironment, such as by inducing changes in PD-L1 expression and immune cell densities [35]. Such findings are consistent with a model in which PD-L1-high EGFR-mutant tumors have measurable but functionally constrained immune engagement, characterized by T-cell dysfunction and enrichment for immunosuppressive programs rather than effective immune-mediated tumor clearance [34,35].

Results from spatially resolved transcriptomic analyses of NSCLC tumor samples stratified by PD-L1 expression further show that PD-L1-high EGFR-mutant tumors exhibit immune and stromal programs distinct from those of PD-L1-low samples, suggesting that PD-L1 status may reflect broader alterations in tissue-level immune architecture rather than a single-marker change [36]. Consistent with this interpretation, immune profiling analyses of samples from osimertinib-treated cohorts have shown that PD-L1-high tumors are associated with IFN-γ-STAT3 activation and enrichment for CD56^bright^ NK cells, suggesting an inflamed but functionally constrained immune microenvironment [16].

**Figure 3:**
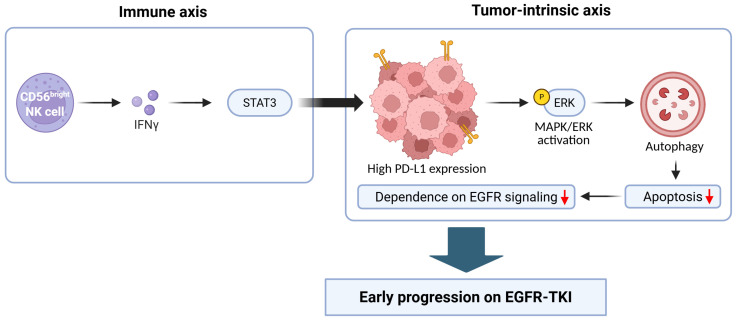
Proposed model linking immune-mediated PD-L1 induction and tumor-intrinsic survival signaling in PD-L1-high EGFR-mutant NSCLC. Interferon (IFN)-γ, produced by CD56^bright^ natural killer (NK) cells, activates signal transducer and activator of transcription 3 (STAT3) signaling and induces PD-L1 expression. The resulting increase in PD-L1 levels promotes mitogen-activated protein kinase (MAPK)/extracellular signal-related kinase (ERK)-dependent autophagy, suppresses apoptosis, and reduces dependence on EGFR signaling, contributing to early progression of patients on EGFR-TKI monotherapy.

Given the above findings, a key mechanistic nuance to consider is that elevated PD-L1 expression in EGFR-mutant NSCLC may arise through more than one biological route, integrating tumor-intrinsic survival programs with immune-derived inflammatory signaling, as summarized in Figure 3. This model is consistent with preclinical work showing that EGFR activation can directly induce PD-L1 expression and facilitate immune escape, which suggests that PD-L1 can act as an oncogene-linked immune evasion feature rather than a pure surrogate of adaptive anti-tumor immune activity [37]. This oncogene-driven immune escape program was originally identified in EGFR-driven lung tumor models, establishing a conceptual foundation for the link between EGFR signaling, PD-1/PD-L1 pathway activation, and immune suppression [10]. In parallel, inflammatory cytokine signaling can amplify PD-L1 expression through pathways such as IL-6/JAK/STAT3, providing a second mechanism for resistance that may reflect inflammatory pressure in the tumor microenvironment [38]. These dual pathways may explain why PD-L1 expression in EGFR-mutant NSCLC does not reliably predict benefit from ICI treatment, as well as why elevated PD-L1 expression occurs within an overall immune milieu that remains ineffective or suppressive.

Importantly, PD-L1 expression in EGFR-mutant NSCLC should not be interpreted as a stable or uniform biological subtype. Rather, it represents a dynamic and context-dependent biomarker that may arise from multiple biological processes. Tumor-intrinsic oncogenic signaling, including activation of the EGFR/STAT3 axis, can directly induce PD-L1 expression, while immune-mediated mechanisms, particularly IFN-γ-driven inflammatory signaling, can further amplify PD-L1 levels. These distinct but overlapping pathways suggest that PD-L1 reflects a composite biological state integrating oncogenic signaling and immune pressure, rather than defining a discrete and stable tumor subtype.

In addition to the above mechanisms, results from experimental models show that resistance to EGFR inhibition can promote immune escape through increased PD-L1 expression and suppression of anti-tumor T-cell activity, indicating that acquired EGFR-TKI resistance can further reinforce immune evasion programs [39]. From a clinical perspective, this finding suggests that PD-L1 upregulation may be both a baseline feature in some tumors and a resistance-associated adaptation in others. Longitudinal analyses of paired tumor samples before and after EGFR-TKI therapy reveal dynamic remodeling of the tumor immune microenvironment, including changes in PD-L1 expression and immune cell composition, supporting a model in which immune escape mechanisms can be reinforced during EGFR-TKI treatment [35]. Thus, both intrinsic processes and acquired adaptations may contribute to reduced benefit from EGFR-TKI monotherapy by coupling tumor cell survival programs with immune suppressive signaling, particularly in biologically heterogeneous tumors that already harbor co-mutations, plasticity programs, or bypass pathway activation [30,39]. Another important consideration is the spatial and temporal heterogeneity of PD-L1 expression in EGFR-mutant NSCLC. From a spatial perspective, PD-L1 expression may differ between primary tumors and metastatic lesions, with emerging evidence demonstrating discordance across anatomical sites. Notably, recent studies suggest that PD-L1 expression in primary tumors may have different clinical relevance compared with metastatic sites, highlighting the complexity of biomarker interpretation. From a temporal perspective, PD-L1 expression is dynamically regulated over the course of disease and treatment. Longitudinal analyses indicate that EGFR-TKI therapy can modulate PD-L1 expression and reshape the tumor immune microenvironment, reflecting adaptive resistance processes. Together, these findings underscore that PD-L1 should be interpreted as a temporally and spatially variable biomarker rather than a fixed tumor characteristic.

Collectively, these molecular and immunologic features provide a plausible mechanistic framework for the observed association between high PD-L1 expression and impaired benefit from EGFR-TKIs. Within this multifaceted context, increased PD-L1 expression has been associated with greater genomic complexity, co-mutational burden, and intrinsic pathway-level alterations [28,29,30]. In parallel, elevated PD-L1 is linked to cell-state plasticity programs, such as EMT [31,32], as well as a distinct immune contexture that is dysfunctional or suppressive, rather than fully quiescent [34,35,36]. Importantly, however, the extent to which PD-L1 expression itself acts as a direct driver of resistance, as opposed to a surrogate marker of a more complex biological state, remains unclear. High PD-L1 expression frequently co-occurs with factors such as TP53 mutations, smoking-related genomic alterations, tumor burden, and inflammatory signaling pathways, all of which may independently contribute to adverse clinical outcomes. Therefore, PD-L1 expression should be interpreted with caution, as it may reflect an underlying constellation of biological features rather than a single causative mechanism. In this context, PD-L1 may function primarily as a biomarker of tumor heterogeneity rather than an independent driver of therapeutic resistance. Further studies are needed to disentangle these relationships and determine the precise role of PD-L1 in EGFR-mutant NSCLC.

In addition to PD-L1-associated heterogeneity, sex-related differences may represent another layer of biological variability in EGFR-mutant NSCLC. EGFR mutations are more frequently observed in female patients, particularly among never-smokers, suggesting potential interactions between hormonal factors and oncogenic signaling. Estrogen signaling has been implicated in modulating EGFR pathway activity and tumor progression in preclinical models. Moreover, sex-based differences in immune responses have been reported, which may influence the tumor immune microenvironment and PD-L1 expression patterns. However, data specifically addressing the role of gender in PD-L1-high EGFR-mutant NSCLC remain limited, and further studies are warranted. These findings suggest that gender may represent an additional layer of biological heterogeneity that warrants further investigation.

Beyond PD-L1, alternative immune checkpoint molecules such as SIGLEC-15 have recently emerged as potential regulators of tumor immune evasion. Unlike PD-L1, which is often associated with adaptive immune resistance and inflammatory signaling, SIGLEC-15 appears to function through distinct immunosuppressive mechanisms and is frequently expressed in tumors with low PD-L1 levels. Notably, PD-L1 and SIGLEC-15 expression have been reported to exhibit a mutually exclusive or non-overlapping pattern in NSCLC, suggesting the existence of parallel immune escape pathways. This raises the possibility that targeting SIGLEC-15 may represent a complementary or alternative immunotherapeutic strategy, particularly in tumors that are less dependent on PD-L1-mediated immune evasion. Incorporating such emerging immune checkpoints into the conceptual framework may help broaden the understanding of immune heterogeneity in EGFR-mutant NSCLC.

Recent advances in single-cell and spatial transcriptomic analyses have provided more direct insights into the tumor immune microenvironment of EGFR-mutant NSCLC. These studies demonstrate that PD-L1-high tumors exhibit distinct spatial organization of immune and stromal components, with enrichment of dysfunctional T-cell states and immunosuppressive signaling networks, supporting the concept of an inflamed but functionally constrained immune microenvironment.

## 5. Therapeutic Implications: Rethinking Treatment Strategies for EGFR-Mutant NSCLC with High PD-L1 Expression

The consistent clinical observation that patients with PD-L1-high EGFR-mutant NSCLC experience earlier progression on EGFR-TKI monotherapy, together with mechanistic signals of reduced EGFR dependency and increased molecular complexity, may suggest the need to explore more risk-adapted therapeutic approaches in this subgroup, which should be evaluated in prospective clinical studies. In practice, the key implication is that PD-L1 level should not be interpreted as an immunotherapy selection marker in EGFR-mutant disease. Rather, high PD-L1 expression may be more clinically useful as a marker of aggressive biology and early resistance risk, prompting consideration of treatment strategies that address tumor heterogeneity and EGFR-independent escape. Importantly, there is currently no prospective evidence supporting PD-L1-driven treatment algorithms in EGFR-mutant NSCLC. Therefore, therapeutic decisions should not be based on PD-L1 expression alone, and its role in guiding treatment selection remains investigational.

A leading option for first-line intensification in patients with PD-L1-high EGFR-mutant NSCLC is osimertinib plus platinum-pemetrexed chemotherapy. In the Phase III FLAURA2 trial, which enrolled patients with EGFR-mutant NSCLC, this regimen improved PFS compared with osimertinib alone [40]. Osimertinib combined with platinum-pemetrexed also provided a significant survival advantage, indicating a durable benefit beyond disease control [41]. Although subgroup analyses stratified by PD-L1 expression were not a prespecified component of this investigation, the results establish a strong proof of principle that upfront multi-modality therapy can outperform EGFR-TKI monotherapy in an unselected EGFR-mutant population [40,41]. Chemotherapy may be particularly beneficial for PD-L1-high tumors that are less EGFR-addicted and more likely to harbor early resistant subclones, as these drugs can suppress EGFR-independent populations and reduce the impact of baseline heterogeneity.

EGFR-directed combination targeted therapy represents another promising first-line intensification pathway. Results from the Phase III MARIPOSA trial showed that amivantamab plus lazertinib improved PFS compared with osimertinib in previously untreated EGFR-mutated advanced NSCLC patients [42]. Although the biologic rationale for this regimen is not PD-L1 driven, the observed efficacy broadens the range of therapeutic options that may be considered for biologically high-risk disease. For PD-L1-high EGFR-mutant tumors, the practical implication is that intensification is no longer limited to adding chemotherapy, and EGFR-directed doublet strategies may represent an alternative route to deepen and prolong disease control, with a distinct toxicity profile [42].

Clinical findings from the pre-osimertinib era identify anti-angiogenic combination strategies as another validated approach to prolong disease control in EGFR-mutant NSCLC. Early evidence was obtained from the JO25567 trial, which reported improved PFS with erlotinib plus bevacizumab compared with erlotinib alone [43]. Similarly, the NEJ026 trial found a PFS benefit with erlotinib plus bevacizumab for patients with EGFR-mutated NSCLC [44]. The RELAY trial further showed that ramucirumab plus erlotinib improved PFS compared with erlotinib alone for those with untreated EGFR-mutated metastatic NSCLC [45]. Although these regimens were developed primarily to enhance the durability of EGFR inhibition rather than to target PD-L1 biology, clinical findings broadly support the premise that biologically higher-risk EGFR-mutant disease may require combination approaches to address non-EGFR tumor dependencies and microenvironmental support.

The potential use of ICIs for treating PD-L1-high EGFR-mutant NSCLC requires careful consideration and sequencing. In general, PD-L1 expression does not reliably predict benefit from immune-checkpoint blockade in EGFR-mutant disease, and immunotherapy should not be used alone due to the elevated levels of PD-L1. Moreover, evidence supporting the use of immunotherapy-containing regimens is strongest in the post-EGFR-TKI setting when ICIs are delivered as part of multi-component combinations. Subgroup analyses from the IMpower150 trial showed benefit with a regimen of atezolizumab plus bevacizumab, carboplatin, and paclitaxel in patients with EGFR mutations, including those previously treated with EGFR-TKIs [46]. Subsequent exploratory reports and follow-up analyses have further described outcomes in EGFR-mutated subgroups and other clinically relevant strata, such as those with liver metastases [47]. In the ORIENT-31 trial, sintilimab plus chemotherapy, with anti-angiogenic backbone components included in the investigational arms, improved outcomes compared with chemotherapy alone after EGFR-TKI failure in EGFR-mutated non-squamous NSCLC [48]. The HARMONi-A randomized trial also showed improved PFS with ivonescimab plus chemotherapy compared with chemotherapy alone in patients with EGFR-variant NSCLC after EGFR-TKI therapy [49]. Collectively, these findings support a pragmatic approach for the use of immunotherapy in patients with EGFR-mutant NSCLC, in which these drugs are administered as part of carefully designed combination regimens after EGFR-TKI resistance, not as monotherapy and not guided by PD-L1 alone.

Safety considerations also strongly influence how immunotherapy can be incorporated into treatment regimens for those with EGFR-mutant NSCLC. Notably, severe immune-related adverse events, particularly pneumonitis, have been reported when osimertinib is administered following PD-(L)1 blockade, indicating that timing and sequencing can materially impact toxicity [50]. This issue is especially relevant in clinical scenarios wherein patients receive immunotherapy after EGFR-TKI progression but may later require osimertinib again through rechallenge strategies or in earlier-line settings if sequencing is unconventional. Accordingly, careful planning, adequate washout, and close monitoring are essential when immunotherapy-based regimens are considered, and strategies to avoid rapid transitions between PD-(L)1 blockade and osimertinib are prudent.

Overall, when considering ICIs for PD-L1-high EGFR-mutant NSCLC, rather than treating these tumors as immunotherapy-responsive analogs of EGFR-wild-type PD-L1-high disease, clinical data suggest that PD-L1-high status should be viewed as a risk signal for early resistance to EGFR-TKI monotherapy. This model provides a rationale for investigating intensification strategies in prospective clinical trials, including osimertinib plus chemotherapy and EGFR-directed combination targeted therapy, as first-line therapies, while reserving immunotherapy-based regimens for evidence-supported post-TKI settings with careful sequencing and toxicity awareness. At present, there is no prospective evidence supporting the use of PD-L1 expression alone to guide treatment selection in EGFR-mutant NSCLC, and therapeutic decisions should not be based solely on PD-L1 status.

## 6. Conclusions and Future Perspectives

The growing body of evidence reviewed herein challenges the long-standing perception of EGFR-mutant NSCLC as a therapeutically uniform entity. In particular, tumors harboring activating EGFR mutations with high levels of PD-L1 expression consistently exhibit clinical and biological features that diverge from the canonical EGFR-driven, TKI-sensitive paradigm. Rather than reflecting immunotherapy responsiveness, elevated PD-L1 expression in this context appears to signify an altered tumor biology characterized by reduced EGFR dependency, increased molecular complexity, and a constrained but non-quiescent immune microenvironment. In total, these features support the hypothesis that PD-L1-high EGFR-mutant NSCLC may represent a biologically distinct phenotype; however, this classification remains exploratory and has not yet been prospectively validated. Importantly, this distinction has implications that extend beyond the choice of individual therapies, underscoring the limitations of single-biomarker decision-making and highlighting the need for integrated classification models that incorporate oncogenic drivers, co-occurring molecular alterations, and features of the tumor-immune microenvironment.

In future studies, this evolving framework also necessitates a shift in clinical trial design. That is, investigators should avoid treating patients with EGFR-mutant NSCLC as a homogeneous group and, instead, incorporate prospective stratification or enrichment strategies based on PD-L1 expression and related biological markers. This approach and the routine reporting of biomarker-defined subgroup outcomes will be crucial for identifying heterogeneous treatment responses and ensuring that clinically meaningful effects are not masked within aggregated trial populations.

Looking ahead, advances in multi-omics profiling, spatial immune characterization, and real-world data integration are likely to further refine risk stratification in EGFR-mutant NSCLC, including the subtype with high PD-L1 expression. Such approaches may enable the identification of patients at risk for early therapeutic failure and inform more precise, biology-driven treatment regimens. Ultimately, recognizing PD-L1-high EGFR-mutant NSCLC as a separate clinical and biological entity represents a critical step toward more nuanced and durable disease control, aligning therapeutic strategies with underlying tumor heterogeneity rather than relying on a uniform treatment paradigm.

## Data Availability

No new data were created or analysed in this study. Data sharing is not applicable to this article.
